# Localized palmar mycosis fungoides: A case report

**DOI:** 10.1097/MD.0000000000047179

**Published:** 2026-01-16

**Authors:** Susu Zhao, Piaopiao Xu, Jianwei Zhu

**Affiliations:** a Department of Dermatology, Quzhou TCM Hospital at the Junction of Four Provinces Affiliated to Zhejiang Chinese Medical University, Quzhou, China; b Department of Dermatology, Hangzhou Meilimei Medical Beauty Clinic, Quzhou, China.

**Keywords:** cutaneous T-cell lymphoma, immunohistochemistry, mycosis fungoides, skin lesions of the palm

## Abstract

**Rationale:**

Mycosis fungoides (MF) is the most common primary cutaneous T-cell lymphoma, yet palmar MF is extremely rare and easily mistaken for chronic dermatoses such as psoriasis. Delayed recognition can lead to inappropriate treatment and worse outcomes. This case demonstrates how careful histopathology and immunohistochemistry can provide critical diagnostic clarity in atypical presentations.

**Patient concerns:**

A 63-year-old woman presented with an 8-year history of erythema, dryness, scaling, and fissuring on both palms. The lesions had been misdiagnosed as psoriasis and showed no response to long-term topical calcipotriol.

**Diagnoses:**

Histopathology of a palm biopsy revealed epidermotropism of atypical lymphocytes with cerebriform nuclei and Pautrier microabscesses. Immunohistochemistry demonstrated positivity for CD2, CD3, CD4, and CD45RO with immunophenotypic variation, confirming palmar MF. The patient was staged as early MF (IA–IB).

**Interventions:**

Systemic therapy with intramuscular interferon α-2b, oral methylprednisolone, and acitretin was initiated due to chronicity, bilateral palm involvement, and resistance to topical therapy.

**Outcomes:**

Significant improvement was noted after 2 months. At the 1-year follow-up, her palms showed complete clearance of lesions with sustained remission.

**Lessons:**

This case highlights the diagnostic pitfalls of palmar MF and underscores the necessity of early biopsy in patients with persistent, treatment-resistant palm dermatoses. Raising clinical awareness of this rare entity may prevent misdiagnosis, facilitate timely therapy, and ultimately improve patient prognosis.

## 1. Introduction

Mycosis fungoides (MF) is the most common from cutaneous T-cell lymphoma, affecting approximately 0.5 per 100,000 individuals annually.^[1]^ The etiology and pathogenesis of MF remain unclear. It typically progresses through 3 stages: macules, plaques, and tumors.^[2]^ Initial skin lesions are frequently erythematous, scaly, or atrophic plaques found in the “bathing trunk” distribution. At later phases, nevertheless, tumors may develop and involve lymph nodes, peripheral blood, and numerous internal organs like the spleen, liver, and gastrointestinal tract.^[3,4]^ Due to its heterogeneous histological features, which vary depending on lesion morphology and the biopsy timing, MF has also been referred to as the “great imitator.”^[5]^ Early MF typically shows epidermotropism of small- to medium-sized cerebriform lymphocytes. However, with the absence of atypical lymphocytes and minimal epidermal infiltration, early MF may be difficult to distinguish from other dermatoses like eczema, psoriasis, and parapsoriasis.^[6]^ Hence, immunohistochemical analysis plays a very important role in differential diagnosis. Malignant T lymphocytes of MF typically express CD3^+^, CD4^+^, and CD8^-^.^[7]^

MF has several rare acral variants that primarily affect the palms and soles. Palmar MF (MF palmaris et plantaris) is 1 such rare variant, presenting with palm or sole involvement. Less than 30 cases have been previously reported. This variant often presents with erythema, scaling, and fissuring, which can mimic chronic dermatitis or psoriasis, making early recognition challenging.^[8]^Another acral variant, pagetoid reticulosis (Woringer–Kolopp disease), is characterized by a solitary, slowly growing scaly plaque containing an intraepidermal proliferation of neoplastic T lymphocytes. This variant differs from classical MF in its clinical presentation and histopathological features.^[9]^ Despite the increasing recognition of these rare acral variants, there remains a knowledge gap regarding the clinical course, diagnostic pitfalls, and optimal management of palmar MF. Therefore, this report aims to emphasize the rare presentation of palmar MF and highlight the importance of early histopathological and immunohistochemical examinations to facilitate accurate diagnosis and appropriate management.

## 2. Case introduction

**Figure 1.**
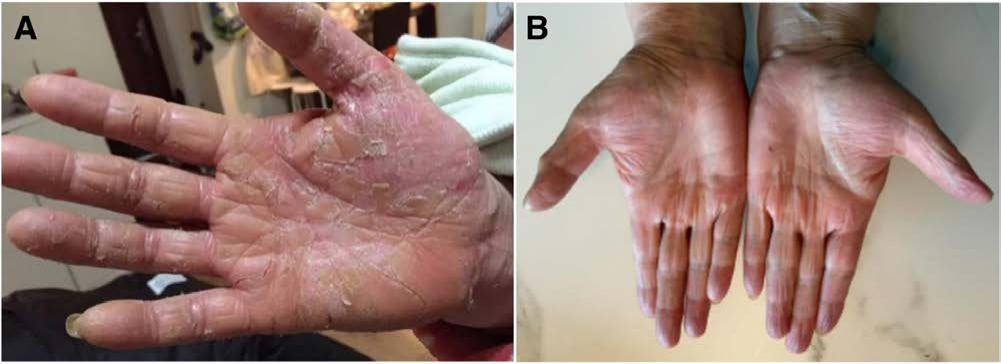
Clinical course of bilateral palmar lesions. (A) Physical examination of bilateral palms showed erythematous, dry, and scaly skin with prominent fissures extending to the wrists. (B) Physical examination showing resolution of bilateral palmar lesions after 1-year treatment with interferon α-2b, methylprednisolone and acitretin.

**Figure 2.**
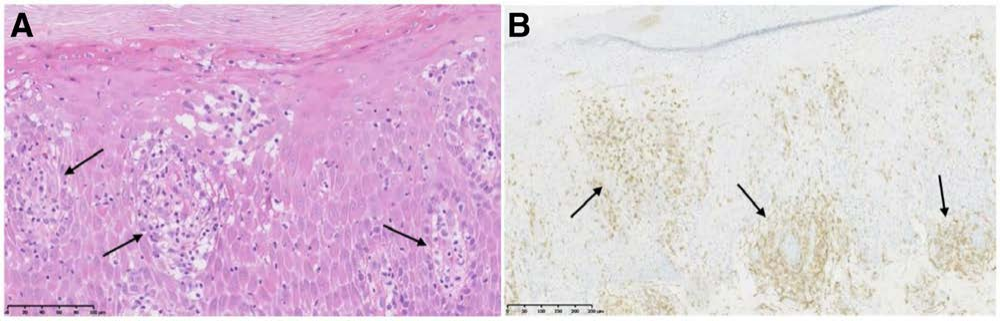
Histopathology and immunohistochemistry of palmar lesions. (A) Lymphocytes epidermotropism with Pautrier microabscesses in the epidermis (arrows). Scale bar = 100 μm. (B) Immunohistochemical staining showing strong CD4 positivity, indicating infiltration of CD4^+^ T lymphocytes (arrows). Scale bar = 200 μm.

A 63-year-old female patient presented to our clinic with an 8-year history of chronic palm skin lesions. There was no significant past medical history except for recurrent exposure of her hands to harsh detergents. She had already been diagnosed with psoriasis and treated with calcipotriol ointment for more than a year and a half. However, due to a lack of clinical improvement, she sought a second opinion at our clinic. Physical examination revealed erythema, dryness, scaling, and fissuring on bilateral palms, extending to the wrists (Fig. 1A). Histopathological analysis of a biopsy from the left palm demonstrated lymphocytic infiltrates characterized by epidermotropism of medium-sized atypical lymphocytes with cerebriform nuclei, along with spongiosis in the spinous layer, and the presence of Pautrier microabscesses (Fig. 2A). In the superficial dermis, these atypical lymphocytes showed nodular hyperplasia with infiltration of the dermal papillae, accompanied by fibrosis, granuloma formation, and infiltration of dermal vasculature and adnexal structures. Immunohistochemical staining showed CD2^+^, CD3^+^, CD4^+^ (Fig. 2B), and CD45RO^+^, consistent with a mature helper T-cell phenotype typical of classic MF. CD5^+^, CD7^+^, CD8^+^, CD20^+^, and CD30^+^ was also observed, indicating immunophenotypic variation.

The negative expression of TIA-1 and Granzyme B indicates the absence of cytotoxic T-cell activity, further supporting the diagnosis of classic MF.^[10]^ Additionally, approximately 40% of the atypical lymphocytes were Ki-67 positive, indicating moderate to high proliferative activity. Based on the clinical presentation, histopathology, and immunophenotype, the patient was staged as early-stage MF (IA–IB).^[11]^ Systemic therapy with intramuscular interferon α-2b, oral methylprednisolone, and acitretin was chosen due to the chronicity, extensive bilateral palm involvement, and failure of topical therapy. Objective evaluation of clinical response included the resolution of erythema, scaling, and fissures on physical examination. Significant symptomatic improvement was noted after 2 months of therapy. At the last follow-up, 1 year after initiating therapy, the patient’s palms showed complete resolution of lesions, with good skin condition. Follow-up was conducted via in-person visits, assessing both clinical signs and patient-reported symptoms to ensure sustained remission (Fig. 1B).

## 3. Discussion

MF is the most common primary cutaneous T-cell lymphoma and may present early as nonspecific eczema-like plaques that are easily overlooked.^[12]^ Misdiagnosis rates in early MF have been reported to be as high as 48%.^[13]^ Palmar MF is a rare variant and may present with atypical features such as localized erythema, fissuring, scaling, or hyperkeratosis, which can mimic chronic dermatitis or psoriasis, making early recognition challenging.^[14]^ In this case, the patient experienced persistent erythema, dryness, scaling, and fissuring of bilateral palms, initially misdiagnosed as psoriasis. The diagnostic delay was likely due to the widespread involvement of the hands, chronicity of lesions, prior topical treatment masking histopathological features, and nonspecific clinical presentation, which together contributed to prolonged misdiagnosis. This highlights the diagnostic complexity of MF, especially at uncommon sites, and the need for increased clinical awareness and timely histopathological evaluation.

Clinically, MF is categorized into 4 stages (stages I–IV), of which stages IA, IB, and IIA belong to the early stage and stages IIB, III, and IV to the advanced stage.^[15]^ Gülseren found that early plaque-stage MF includes epidermal convergence of small heterogeneous lymphocytes, papillary intradermal patchy or banded infiltrates, and occasional Pautrier microabscesses. Comparison with previously reported palmar MF cases suggests that atypical histopathological features, including fibrosis or granuloma formation, are more common in chronic or localized palmar involvement. As the disease progresses, chronic fibrotic changes such as filamentous collagen bundles and perilymphocytic halos become more prominent.^[13]^ In the advanced stages, tumor formation, erythroderma, and histologically confirmed lymph node or visceral involvement are common.^[16]^ In this case, histopathology showed complex features including epidermotropism of medium-sized atypical lymphocytes with cerebriform nuclei, spongiosis in the spinous layer, Pautrier microabscesses, nodular hyperplasia of atypical lymphocytes infiltrating the dermal papillae, fibrosis, granuloma formation, and dermal vasculature and adnexal infiltration. Such atypical clinical and histopathological features may be related to chronicity, extensive hand involvement, or individual variation, reflecting the heterogeneity of MF presentations. These findings indicate that palmar MF may present with advanced or atypical histological features even in the absence of systemic involvement.

The expression pattern of CD markers plays an important role in the diagnosis of MF, although it may vary due to disease progression or tumor heterogeneity. Alnasser et al reported that malignant T lymphocytes in classic MF exhibit CD3^+^, CD4^+^, CD45RO^+^, and CD8^−^ immunophenotype. However, approximately 20% of early-stage MF cases may present the CD8^+^ phenotype, and loss of CD2, CD5, and/or CD7 expression within the lesion or confined to the epidermis is considered highly specific for MF.^[17]^ Khunger et al found that stage IB MF retains CD2^+^, CD3^+^, CD4^+^, CD5^+^, CD7^+^, and CD8^−^ immunophenotype.^[18]^ Wu et al noted that strong CD30 expression is usually absent in MF but may be present on large cells in cases where MF has undergone transformation into high-grade large cell lymphoma.^[19]^ Moreover, Shark et al reported that CD20-positive MF cases are rare, and may be associated with disease progression and more aggressive phenotype.^[20]^ In our case, immunohistochemical staining showed CD3^+^, CD4^+^, and CD45RO^+^, consistent with the phenotype of mature helper T cells in classic MF. However, we also observed CD2^+^, CD5^+^, CD7^+^, CD8^+^, CD20^+^, and CD30^+^, indicating immunophenotypic variation even in early-stage or variant MF. This atypical immunophenotype highlights the importance of comprehensive immunohistochemical evaluation, especially in chronic, treatment-resistant, or atypical. Granzyme B and TIA-1 are cytotoxic proteins typically expressed by cytotoxic CD4⁺ or CD8⁺ T cells. In this case, the absence of TIA-1 and Granzyme B expression further supporting a helper T-cell phenotype of classic MF.^[21]^ Studies have shown that Ki-67 expression is significantly elevated in cases of large cell transformation compared to non-transformed MF.^[22]^ The relatively high Ki-67 index in this patient suggests increased proliferative activity, which may indicate a more aggressive disease course and warrants careful clinical follow-up.

As a case report, this study has several limitations. Observations from a single patient may not be generalizable, and long-term outcomes remain unknown. Moreover, delayed recognition and prior treatment may have influenced histopathological and immunophenotypic findings. Nevertheless, this case, emphasize the importance of recognizing atypical presentations of MF, especially at uncommon sites like the palms, and performing early biopsy for treatment-unresponsive or chronic lesions. Large-scale cohort studies will be necessary in the future to better characterize the clinical, histopathological, and immunophenotypic spectrum of palmar MF and guide management strategies.

## 4. Conclusion

The report presents a rare case of palmar MF with a focus on diagnostic difficulty due to its unpredictable and atypical clinical presentations. Histopathology and immunohistochemistry are key investigations to enhance diagnostic accuracy. This case underscores the importance of considering MF in patients initially diagnosed with psoriasis or other chronic dermatoses that do not respond to conventional treatment. Immunohistochemistry plays a crucial role in confirming the diagnosis, especially in treatment-resistant or atypical cases. Early and accurate diagnosis, combined with individualized therapy, is essential for improving patient prognosis. Physicians should maintain a high index of suspicion for MF in patients with chronic and atypical skin lesions. Timely completion of full diagnostic workups is required to confirm the diagnosis and for guiding individualized treatment approaches.

## Author contributions

**Conceptualization:** Jianwei Zhu.

**Data curation:** Susu Zhao, Piaopiao Xu.

**Funding acquisition:** Jianwei Zhu.

**Investigation:** Susu Zhao.

**Writing – original draft:** Susu Zhao.

**Writing – review & editing:** Jianwei Zhu.
